# Are Entry Criteria for Cataract Surgery Justified?

**DOI:** 10.1371/journal.pone.0112819

**Published:** 2014-11-17

**Authors:** Daniel Böhringer, Werner Vach, Kai Hagenlocher, Philipp Eberwein, Philip Maier, Thomas Reinhard

**Affiliations:** 1 Eye Center, University Hospital, Freiburg, Germany; 2 Center for Medical Biometry and Medical Informatics, Medical Center - University of Freiburg, Freiburg, Germany; Medical University Graz, Austria

## Abstract

**Purpose:**

The German Ophthalmological Society (GOS) recently proposed surgical entry criteria, i.e. 300 cataract surgeries. We herein correlate the surgical hands-on experience with the risk of posterior capsule ruptures in order to assess whether this number is appropriate.

**Methods:**

We identified all cataract operations that had been performed at the University Eye Hospital Freiburg since 1995. For each surgeon, we assigned a running number to his/her procedures in the order they had been performed. Thereafter, we excluded all combined procedures and the second eyes. We then selected the 5475 surgical reports between November 2008 and November 2012 for detailed review. We additionally classified each surgery into low- vs. high- à priori risk for posterior capsule ruptures. We fitted a multifactorial logistic regression model to assess the GOS recommendation of 300 surgeries under supervision. In the low-risk group, we additionally visualized the 'typical' learning curve by plotting the posterior capsule ruptures against the respective rank numbers.

**Results:**

The odds ratio for posterior capsule ruptures of 'learning-mode' (one of the respective surgeon's 300 first procedures) vs. the non-learning-mode was 3.8 (p<0.0001). By contrast, classification into the low-risk group lowered the risk of posterior capsule ruptures three fold (p<0.0001). According to the low-risk plot, the surgeons started with a complication rate of 4% and continuously improved towards 0.5% after 1500 operations. Thereafter, the rate increased again and stabilized around one percent.

**Conclusion:**

The learning curve with respect to posterior capsule ruptures is surprisingly flat. The GOS entry criterion of 300 cataract procedures is therefore most likely justified. Careful selection of low-risk patients for the training surgeons may help in reducing the rate of posterior capsule ruptures during training.

## Introduction

Entry criteria in the context of surgical quality management are highly controversial because they are commonly regarded as an act of patronisation. This especially holds in the lucrative field of cataract surgery. Consequently, a common counter-argument is the lack of evidence that entry criteria can improve long-term outcomes [1]. Nevertheless, a beneficial training-effect is documented. However, this evidence is mostly cross-sectional [2]–[4] and thus subject to a multitude of confounding factors.

By contrast, individual learning curves have been only casually analyzed [5]–[7]. This places the recent proposal of the German Ophthalmological Society (GOS) in a tenuous position: the GOS recently recommended, that the first 300 cataract surgeries should generally be performed under supervision. We herein assess in large longitudinal monocentric retrospective analysis, whether this threshold of 300 cataract procedures is appropriate or too high. We focus on posterior capsule ruptures because this is the most common complication with sight-threatening implications in modern cataract surgery [8], [9].

## Results

We observed a total of 100 posterior capsule ruptures in our cohort. The percentage was expectedly higher in the high-risk group in comparison to the low-risk group (5% vs. 2%). In our multifactorial logistic regression analysis, both the training classification and the classification into the high- vs. low-risk group turned out statistically significant influencing factors on the risk of posterior capsule ruptures (p<0.001).

**Figure 1:**
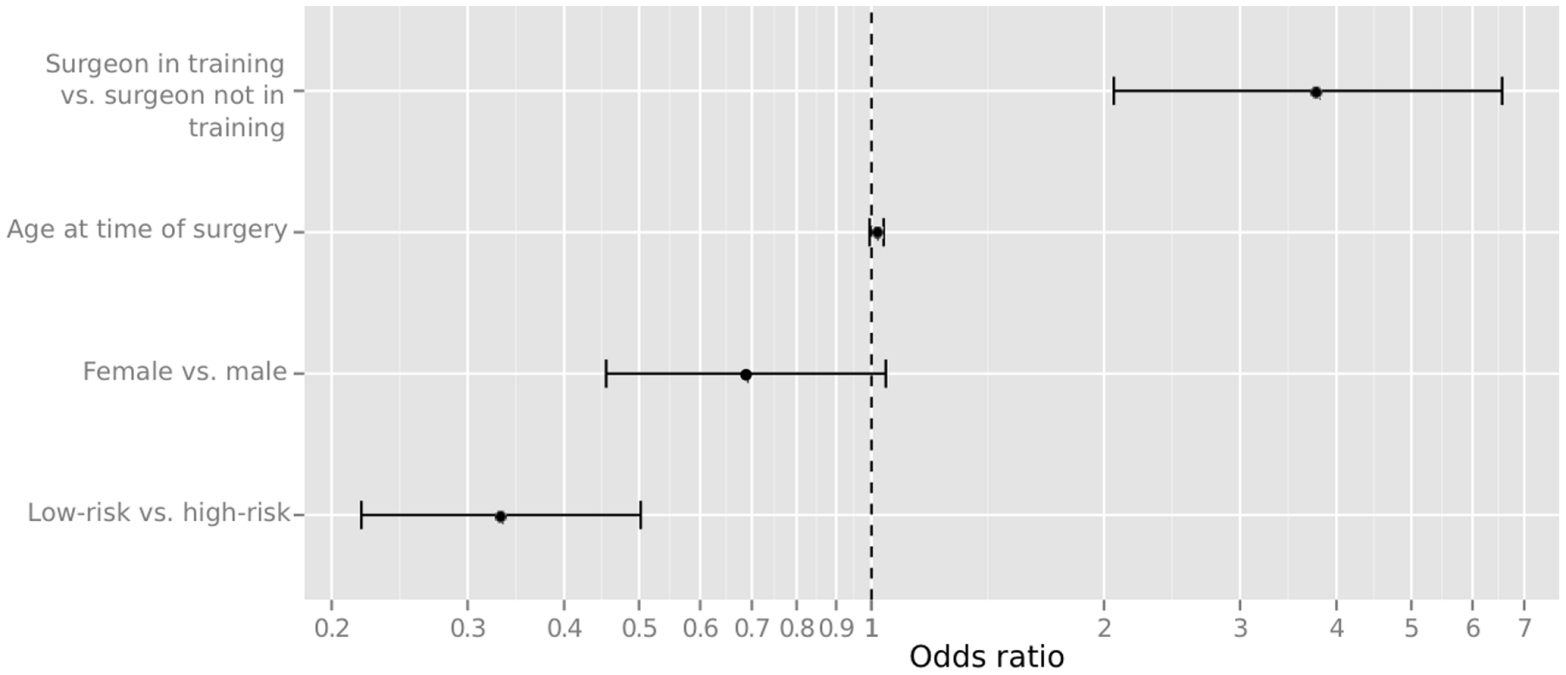
Odds ratios for posterior capsule ruptures from our logistic regression model.

A training surgery is 3.8 times more likely to experience a posterior capsular break in comparison to a surgery with a more experienced surgeon. The opposite it true for the low- in comparison to the high-risk classification. Low-risk operations are three times less likely to be affected from a posterior capsule ruptures. We also observe a tendency for elder patients experiencing more posterior capsule ruptures whereas female gender seems to be associated with a reduced risk of capsular ruptures. Both factors miss statistical significance, though. The odds ratios from our logistic regression model are depicted in figure 1.

**Figure 2:**
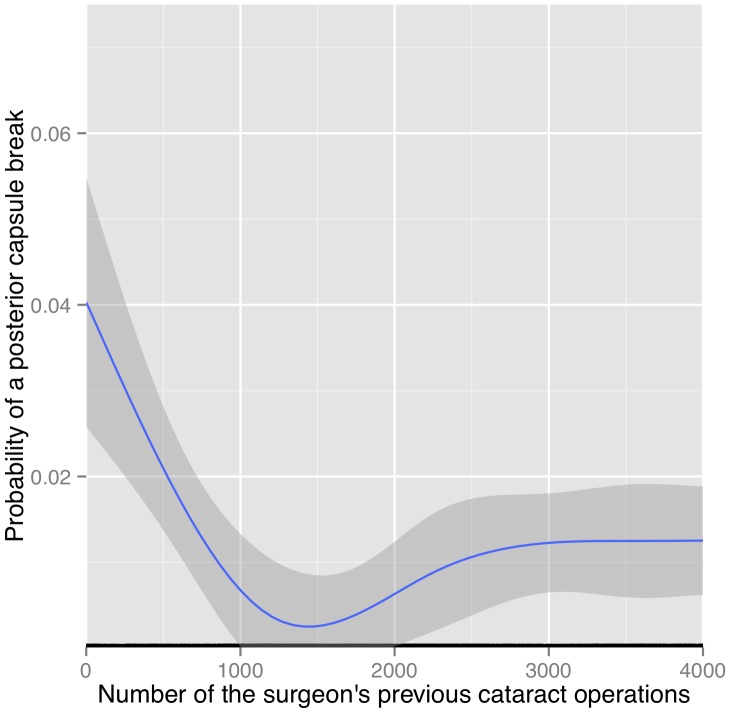
Natural spline fit with three degrees of freedom after plotting the posterior capsule ruptures against the respective rank numbers. The grey ribbon denotes the standard error of the mean.

We also aimed at analyzing the typical learning curve in cataract surgery in addition to assessing the GOS criterium of 300 operations under supervision. We therefore selected the low-risk group in order minimize all potential confounders as much as possible. We generated a scatterplot of capsular rupture coded as one vs. no capsular break coded as zero against the surgeon-specific rank number of the respective operation. A spline-fit through this plot can be interpreted as the probability for posterior-capsule ruptures as a function of the surgeons's experience. This graphical analysis is depicted in figure 2. A novice surgeon typically starts with a posterior capsule break rate of 4%. We observe a linear improvement towards zero posterior capsule ruptures after approximately 1500 procedures. Thereafter, the rate increased and stabilized around one percent.

## Discussion

Our primary goal was to assess whether the GOS proposal of 300 cataract procedures is appropriate or too high. We focussed on posterior capsule ruptures as a proxy for the overal complication rate. This makes sense because posterior capsule rupture is the single most common complication in modern cataract surgery [9]. Avoiding posterior capsule ruptures is essential because they may give rise to retinal detachment, cystoid macular oedema and eventually a higher overall likelihood of permanently decreased visual acuity [8]. Furthermore, posterior capsule ruptures can be unambiguously identified from the medical records.

We selected a large homogenous cohort with a only few cataract surgeons at all stages of the indivdual career. We are not aware of any equally large investigation on the longitudinal learning curve in cataract surgery. Our major finding was a relevant and statistically significant reduction in the rate of posterior capsule ruptures after getting through the first 300 procedures. This was somehow expected because a training effect in cataract surgery has been reported in the literature. However, the current evidence suggested much steeper learning curve. E.g. Randleman et al report a significant reduction from 11% down to 5% in posterior capsule ruptures in the first 160 resident cases [7]. Tarbet et al. report similar numbers [6] whereas Lee et al. even postulate an exponential learning curve [10]. By contrast, in our rather unbiassed low-risk group, the rate of posterior capsular ruptures steadily fell over the first 1500 procedures (figure 2). It is thus likely, that the aforementioned much smaller investigations stopped before the learning curve leveled off.

The longitudinal learning-curve data of three trainees from an intensive cataract surgery training program in the United Kingdom (UK) have recently been published [5]. There, the posterior capsule break rate lowered from 1% to 0.66% only after 291–318 cataract procedures. However, our initial rate of posterior capsule ruptures of 4% is higher than the 1% reported in the three UK trainees. This may be due to the extensive simulator training over two years in this particular training program. Another large study from the UK confirms the negative correlation of the surgeon's experience with the rate of posterior capsule ruptures [3]. Here, the rate of posterior capsule ruptures drops from 2.5% in the first year to 1.3% in the third year but rises again back to 2% thereafter. We also observe the resurgence of posterior capsule ruptures in the more experienced surgeons (figure 2). We have no good explanation for this phenomenon, but overestimation of one's own capabilities or weakening vigilance come to mind. However, we did not observe any particular accumulation of incidents over the operating room day (data not shown).

In conclusion, the GOS proposal of 300 operations is most likely not too high. This threshold is rather basely because the learning phase may continue for much longer than 300 operations under supervision. However, careful selection of low-risk patients for the training surgeons may help in reducing the rate of posterior capsule ruptures during the training period.

## Materials and Methods

We reviewed all surgical protocols of the University Eye Center Freiburg since 1995. For each surgeon, we assigned a running number to the cataract operations in the order they had been performed. We used this lifetime number of the surgeon's preceding cataract operations to operationalize his surgical experience. We then selected a cohort of 13379 cataract surgeries that had been performed between November 2008 and November 2012. Thereafter, we excluded all combined procedures and the second eyes. This resulted in a large group of 5475 statistically independent cataract operations for detailed manual review. All data were anonymized and de-identified prior to analysis. This study has been conducted according to the principles expressed in the Declaration of Helsinki. The ethics committee of the Albert-Ludwigs-University of Freiburg approved our study (approval number: 471/12).

We carefully reviewed the surgical reports for the mentioning of posterior capsule ruptures. We additinonally classified each surgery into a low- vs. high-risk group. This was done on the basis of all data that was evident prior to surgery. Low-risk was defined as the absence of the following criteria in the medical records predating surgery: pseudoexfoliation, corneal opacification, prior keratoplasty, shallow anterior chamber, phakodonesis and very dense cataract. The first 300 cataract operations of each surgeon were eventually classified as ‘training surgery’ in agreement with the aforementioned GOS proposal. This classification was performed only after all other data had been entered in order to control for any coding-bias. The patient characteristics are summarized in table 1.

**Table 1:** Patient characteristics of our groups.

	Surgeon not in training	Surgeon in training
	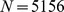	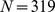
Patient age at time of surgery (+/− standard deviation)	75+/−11	77+/−12
Percentage females	56% _(2890)_	62% _(199)_
Percentage low-risk	73% _(3746)_	79% _(252)_
Cumulative incidence of capsular ruptures	2% _(84)_	5% _(16)_

The first 300 surgeries of each surgeon are classified as 'surgeon in training' whereas all following procedures are classified as 'surgeon not in training'. Absolute numbers are given in backets.

We entered all data into dedicated electronic case report forms. Our ethics committee approved the study. We adhered to the tenets of the Declaration of Helsinki in this non-interventional retrospective investigation.

### Surgical technique and training

All operations had been performed using the small incision clear cornea phakoemulsification technique. At the University Eye Center, trainees are generally supervised by an experienced surgeon. After the first procedures, this supervision is then progressively eased at the discretion of the supervisor. The median time period for the first 300 surgeries was 631 days (upper and lower quartiles were 272 and 967, repectively). The surgeons' surgical experience is summarized in table 2.

**Table 2:** Surgeon's experience as operationalized by the number of cataract operations already performed at the beginning and the end of our investigation.

Surgeon	Number of cataract surgeries in 2008	number of cataract surgeries in 2012
Surgeon 1	3276	4484
Surgeon 2	1672	2947
Surgeon 3	1	21
Surgeon 4	36	1761
Surgeon 5	1437	1649
Surgeon 6	5	177
Surgeon 7	1	1277
Surgeon 8	1	1496
Surgeon 9	859	3325
Surgeon 10	3134	4414
Surgeon 11	2090	2636
Surgeon 12	7	221
Surgeon 13	3365	5285

### Statistical analyses

We fitted a multifactorial logistic regression model to describe the risk of posterior capsule ruptures with the surgeon's training state and the low- vs. high-risk classification. We also included age at time of surgery and gender as co-variates. In the low-risk group, we additionally spline-fitted the learning curve after scatter-plotting the posterior capsule ruptures against the surgeons' cumulative experience in cataract surgery. We performed the calculations with R and Stata.

## Supporting Information

Data S1 **Dataset of all cataract surgeries from this manuscript.** The data are tabulator delimited. Each row comprises a single observation.
(ZIP)
